# Supplementary material for: 3D Printing of Thermal Insulating Polyimide/Cellulose Nanocrystal Composite Aerogels with Low Dimensional Shrinkage
Source: Polymers (Basel). 2021 Oct 20;13(21):3614. doi: 10.3390/polym13213614 (PMC8588507; doi:10.3390/polym13213614)
Supplement: Supplementary file 1 [file polymers-13-03614-s001.zip › polymers-1420727-supplementary.pdf]

Supplementary Material

3D Printing of Thermal Insulating Polyimide/Cellulose Nanocrystal Composite Aerogels with Low Dimensional Shrinkage

*Chiao Feng and Sheng-Sheng Yu**

Department of Chemical Engineering, National Cheng Kung University, Tainan 70101, Taiwan (R.O.C)

Core Facility Center, National Cheng Kung University, Tainan 70101, Taiwan

Program on Smart and Sustainable Manufacturing, Academy of Innovative Semiconductor and Sustainable Manufacturing, National Cheng Kung University, Tainan 70101, Taiwan

*Email: ssyu@mail.ncku.edu.tw

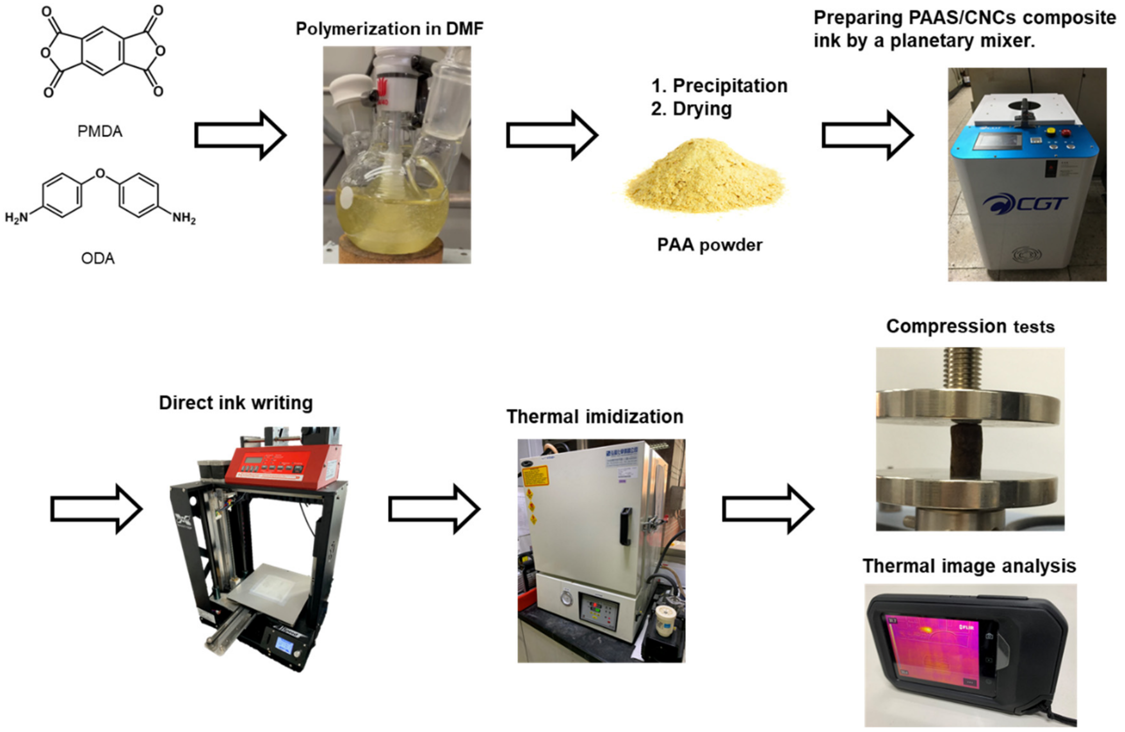

Figure S1. Synthesis and characterization of the 3D printable PI/CNCs composite aerogels.

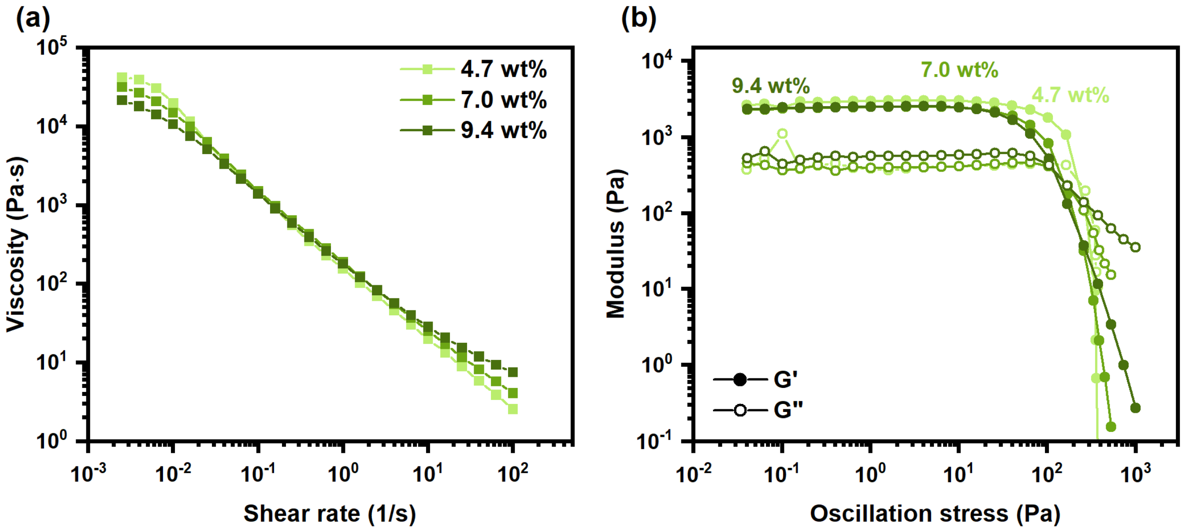

Figure S2. Rheological analysis of the inks under different concentrations of polyamic acid. (**a**) Steady-state shear viscosity of the ink. (**b**) Oscillatory rheological measurement.

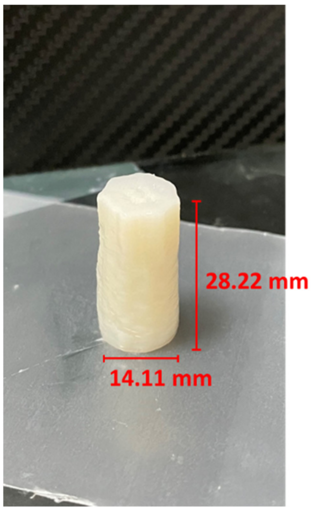

Figure S3. A 3D printed cylinder that shows the thickness achievable by the PAAS/CNCs ink. The concentrations of PAAS and CNCs were 7.0 wt% and 6.5 wt%, respectively.

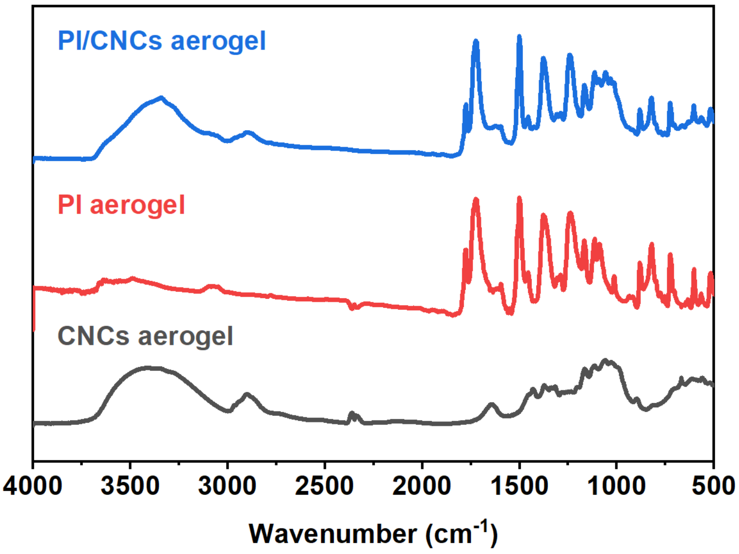

Figure S4. Full FT-IR spectra of different aerogels.

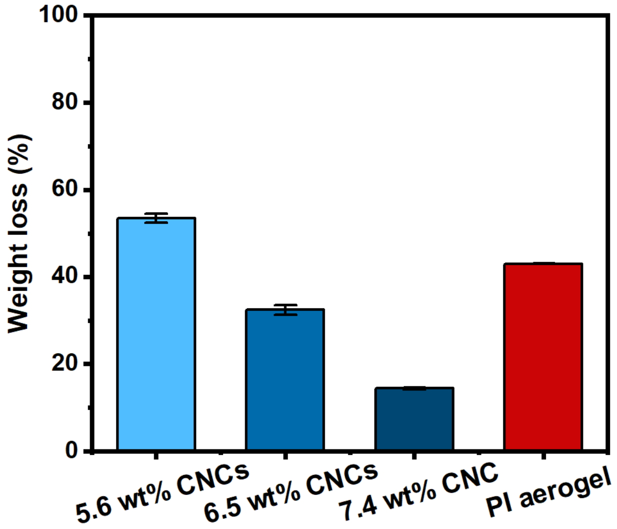

Figure S5. Weight loss of the freeze-dried samples after thermal imidization.

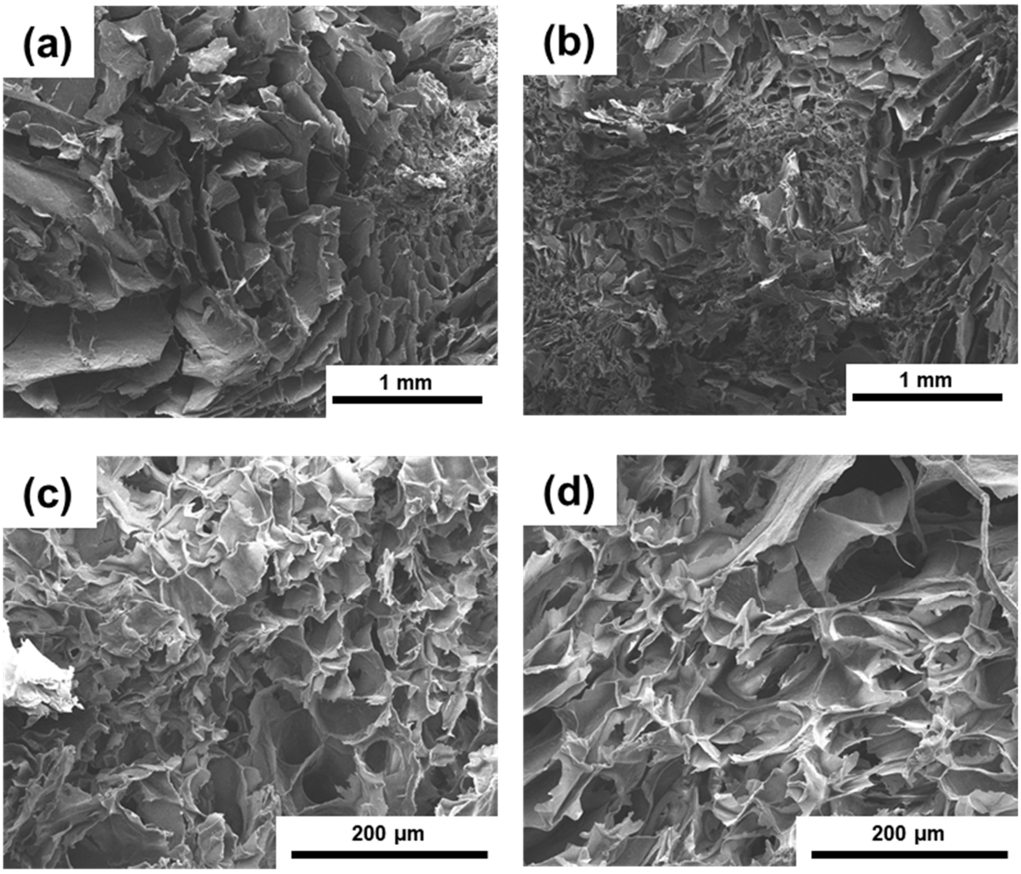

Figure S6. Cross-sectional SEM images of the composite aerogels after the thermal imidization process. (**a**,**c**) 5.6 wt% CNCs. (**b**,**d**) 7.4 wt% CNCs.

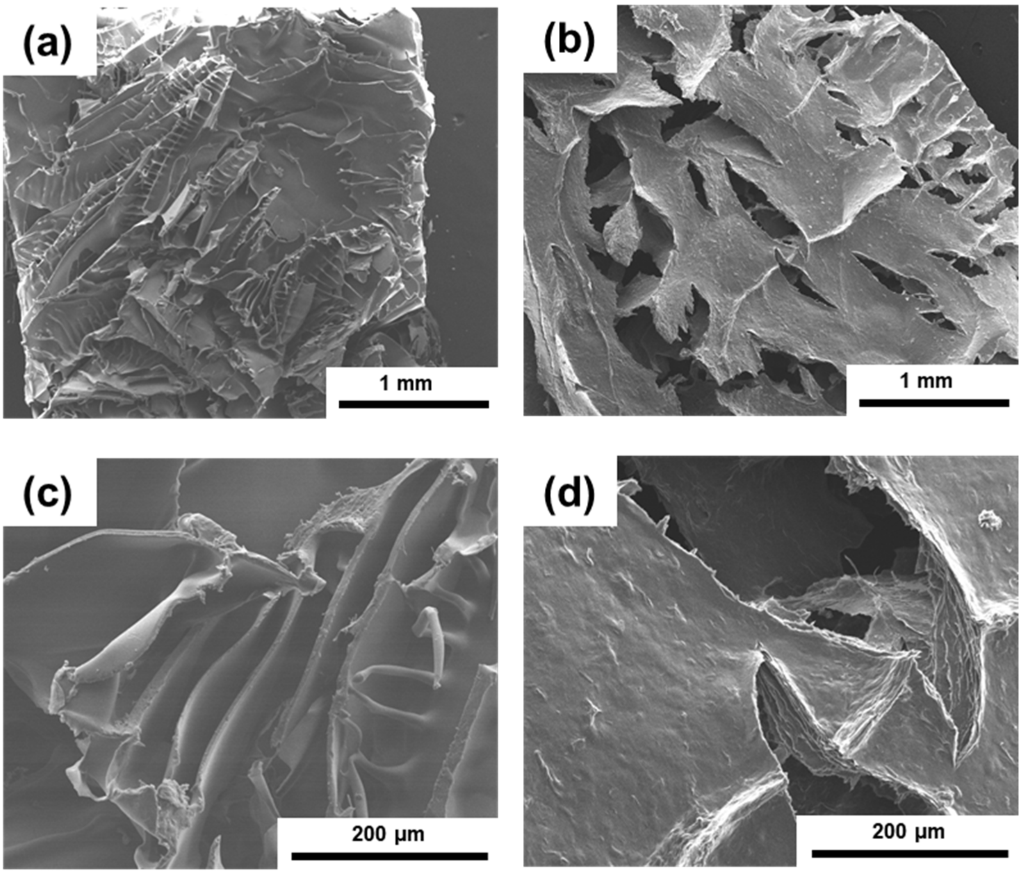

Figure S7. Cross-sectional SEM images. (**a**,**c**) Pure PAAS aerogels. (**b**,**d**) Pure CNC aerogels after freeze-drying.

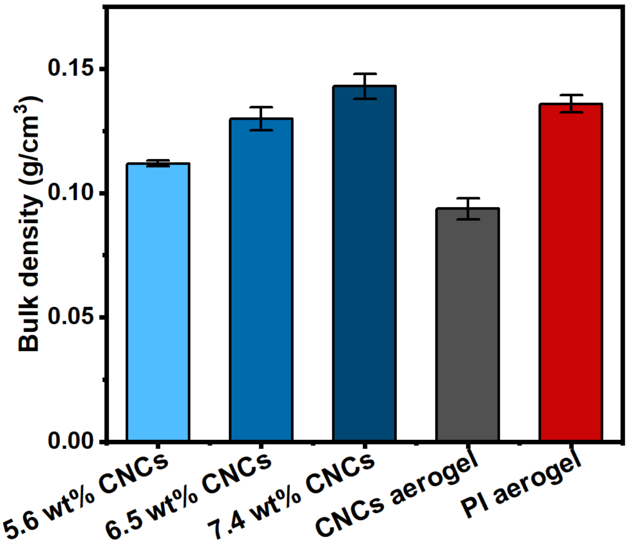

Figure S8. Bulk densities of PI/CNCs composite aerogels, CNCs aerogels, and PI aerogels.
